# CBCT analysis of crestal soft tissue thickness before implant placement and its relationship with cortical bone thickness

**DOI:** 10.1186/s12903-022-02629-w

**Published:** 2022-12-10

**Authors:** Xiaoxi Cui, Tyler Reason, Vanessa Pardi, Qiang Wu, Acela A. Martinez Luna

**Affiliations:** 1 Department of General Dentistry, School of Dental Medicine, East Carolina University, Greenville, NC USA; 2 School of Dental Medicine, East Carolina University, Greenville, NC USA; 3 Department of Foundational Sciences, School of Dental Medicine, East Carolina University, Greenville, NC USA; 4 Department of Public Health, Brody School of Medicine, East Carolina University, Greenville, NC USA; 5 Division Director of Clinical Implantology, Department of Surgical Sciences, School of Dental Medicine, East Carolina University, Greenville, NC USA

**Keywords:** Crestal soft tissue thickness, Dental implants, Cone beam computed tomography

## Abstract

**Background:**

The importance of crestal soft tissue thickness and its influence in peri-implant tissue health has been evaluated in few clinical studies. Cone beam computed tomography imaging offers a unique opportunity to investigate variations in crestal soft tissue thickness. The aim of this retrospective study was to evaluate the possible correlation between crestal soft tissue thickness and hard tissue measurements on CBCT images, and to compare crestal soft tissue thickness among different patients and edentulous site groups.

**Methods:**

CBCT images of partially edentulous adult patients treated at ECU School of Dental Medicine were evaluated. 267 patients with 321 edentulous sites were included. Demographic data were collected from electronic health records. Cross-sectional CBCT images at the center of each edentulous site were used to measure soft tissue and hard tissue parameters. Linear mixed models were used to compare crestal soft tissue thickness and hard tissue measurements by gender, age groups, and edentulous sites. Pearson correlation was applied to evaluate the correlation between crestal soft tissue thickness and different hard tissue measurements. Association between crestal soft tissue thickness and independent variables (gender, age groups, edentulous sites) was evaluated using repeated measure logistic regression, while the crestal soft tissue thickness was dichotomized by a threshold of 2 mm.

**Results:**

Mean age of patients included was 60 (range 21–85 years). Female to male ratio was 1.07. Mean crestal soft tissue thickness of all non-grafted native bone sites was 2.17 mm. Mean thickness of cortical bone at alveolar crest was 0.94 mm. Thickness of buccal and lingual cortical plates 5 mm apical to alveolar crest were 1.17 mm and 1.58 mm, respectively. Pearson’s correlation showed moderate positive correlation among hard tissue measurements, but weak correlation between soft tissue thickness and hard tissue measurements. Anterior sites [OR = 3.429 (1.100–10.69)] and maxillary posterior sites [OR = 1.937 (1.077–3.482)] had higher odds of presenting with more than 2 mm of soft tissue at the alveolar crest.

**Conclusion:**

More than half of the patients had crestal soft tissues at edentulous sites thicker than 2 mm. Thickness of crestal soft tissue was not significantly associated with hard tissue measurements. Edentulous anterior sites and maxillary posterior sites presented with thicker crestal soft tissue at alveolar crest as compared to mandibular posterior sites.

## Background

The use of dental implants to support prostheses is a widely accepted treatment modality of high success and predictability. The importance of peri-implant bone stability for the success and longevity of treatment has always been emphasized [1]. Various factors, such as implant design and surface configuration [2, 3], platform switching [4], implant insertion depth and abutment height [5], occlusal loading [6], and the amount of soft‐tissue volume and keratinized tissue [7], have been reported to be crucial for predictable peri-implant crestal bone stability. Among them is initial crestal soft tissue thickness. In 1996, Berglundh and Lindhe first demonstrated in an animal study that a certain minimum thickness of peri-implant mucosa was necessary to establish stable soft tissue attachment around dental implants [8]. Recent clinical studies and systemic reviews have also shown that thick initial mucosa (> 2 mm) surrounding implants is associated with significantly less crestal bone change in the short term [9–12].

Crestal soft tissue can also be called “vertical soft tissue.” To date, most of the methods for measuring vertical soft tissue width have focused on direct physical measurement with surgical manipulation. Linkevicius et al. [13] reported a clinical method used to measure crestal soft tissue thickness prior to dental implant placement. After the administration of local anesthesia, a full-thickness buccal flap was raised, and the thickness of the unseparated lingual flap was measured with a periodontal probe at the alveolar crest in the center of future implant placement. Jeong et al. [14] described another method to measure soft tissue thickness where flapless implant placement was planned. They used a 3 mm soft tissue punch at the proposed implant site, removed the core soft tissue from over the crestal bone, then measured the crestal soft tissue thickness using a periodontal probe. However, both methods were invasive and were applied shortly prior to implant placement surgery. Thus, accurate measurement of crestal soft tissue thickness was unknown until the day of implant surgery.

The development of radiological imaging in the form of cone-beam computed tomography (CBCT) provides a non-invasive, precise demonstration of anatomical structures and has been proved extremely useful in dentistry due to its relatively low exposure dose and high resolution [15, 16]. CBCT has become increasingly utilized in presurgical evaluations of jawbone quality and quantity [17]. Although CBCT was initially an exclusive tool for hard maxillofacial tissue imaging, it has been used to analyze soft tissue in facial area in the last decades. Studies have proved that CBCT can be a precise and noninvasive method for soft maxillofacial tissue imaging [18–21]. Despite the vast scientific literature on the importance of crestal soft tissue thickness to peri-implant bone stability, no study was designed to investigate variations of crestal soft tissue thickness and its correlation to hard tissue measurements using non-invasive CBCT images.

Therefore, the primary objectives of this study were to use CBCT images to evaluate thickness of crestal soft tissue of both bone grafted and non-grafted edentulous sites ready for implant placement, and to compare crestal soft tissue thickness among different patient groups and different edentulous sites. The secondary objectives were to determine possible correlation between alveolar hard tissue measurements and crestal soft tissue thickness, and association between crestal soft tissue thickness and independent variables (gender, age groups, edentulous sites) in non-grafted native bone sites.

## Methods

This retrospective study was approved by East Carolina University (ECU) institutional review board (UMCIRB 20-000945). Dental records of partially edentulous adult patients (≥ 18 years old) who were treated with single implant supported crowns at Comprehensive Care Clinic of ECU School of Dental Medicine from March 1, 2014 to March 1, 2022 and had at least one CBCT image taken prior to implant placement were reviewed. Inclusion criteria were: (1) generally healthy patients, no medical contraindication for implant surgery; (2) patient’s single implant site was adjacent to two natural teeth or distal to one natural tooth if the implant site was the most distal site of the arch; (3) patient’s CBCT image showed measurable crestal soft tissue at the implant site. Patient’s records were excluded if they did not meet the inclusion criteria and if they additionally (1) had soft tissue graft surgery at the implant site before taking CBCT; (2) had medical conditions or were taking medications that can affect bone or soft tissue wound healing, such as uncontrolled diabetes mellitus or osteoporosis, or using bisphosphonates. Patients who had socket preservation surgery or other bone grafting procedure before taking CBCT were included but no hard tissue related measurements were analyzed. The patient-related factors collected from patients’ dental records were: (1) age; (2) gender; (3) ethnicity; (4) edentulous site planned for implant placement.

### CBCT imaging acquisition and measurement

Two types of CBCT units were used, Instrumentarium OP300 (KaVo Dental Excellence, Biberach, Germany) and Sirona Orthophos SL (Dentsply Sirona, Charlotte, NC, USA). The scans taken with Instrumentarium OP300 were acquired at 89.8 kV and 6 mA, 13 s, 0.3 mm^3^ voxel size, and signal grey scale was 16-bit. Field of view (FOV) of scans used in this study was 60 × 80 mm^2^. The scans taken with Sirona Orthophos SL were acquired at 85 kV and 10 mA, 14 s, 0.2 mm^3^ voxel size, and signal grey scale was 16-bit. FOV of scans used in this study was 80 × 80 mm^2^. All images were displayed on a MacBook Pro (16-inch, 2019) laptop computer (Apple, Cupertino, CA, USA) equipped with graphic card 2048 MB, 2560 × 1440 pixels.

**Fig. 1:**
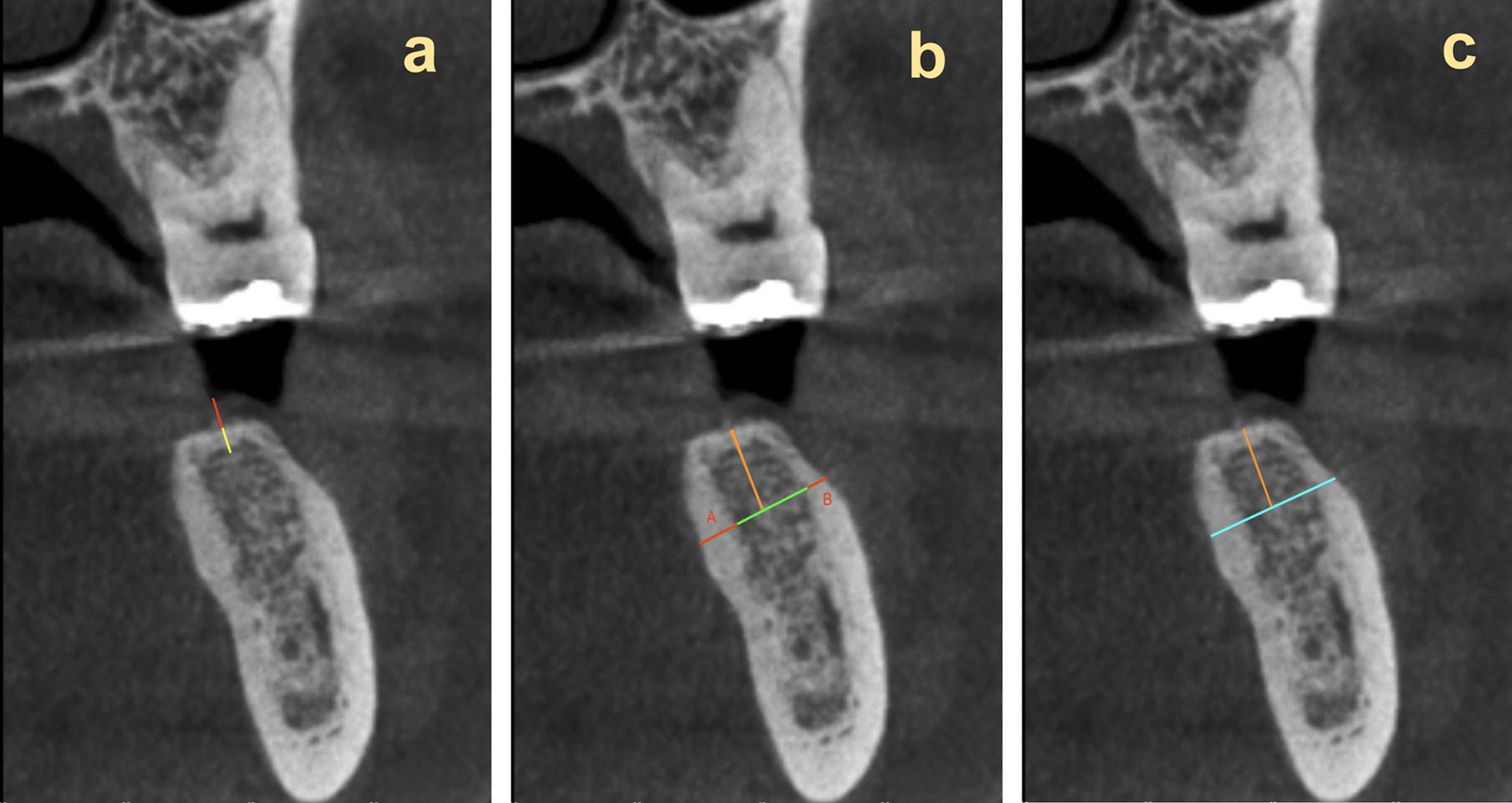
Measurements evaluated on CBCT images **a** Crestal soft tissue thickness measured in red and cortical bone of alveolar crest measured in yellow. **b** 5 mm apical to alveolar crest shown in orange. Lingual cortical plate (A) and buccal cortical plate (B) thickness measured in red. **c** 5 mm apical to alveolar crest shown in orange. Alveolar ridge thickness measured in blue (including thickness of buccal and lingual cortical plates)

Vertical cross-section views perpendicular to the alveolar ridge at the center of each edentulous site were reviewed and measured using Invivo 6™ Software (Anatomage, San Jose, CA, version 6.0.3). Measurements evaluated on CBCT images were as follows (Fig. 1 Measurements evaluated on CBCT images): (1) thickness of crestal soft tissue at the alveolar crest; (2) thickness of cortical bone at the alveolar crest; (3) thickness of buccal and lingual cortical plates 5 mm apical to the alveolar crest; and (4) width of alveolar ridge (from buccal cortical plate to lingual cortical plate) 5 mm apical to the alveolar crest. Units were measured in millimeters.

One general dentist (X.C.) was trained and assessed CBCT scans from 10 randomly selected patients for calibration, measuring the factors listed above. The measurements were repeated one week later with the initial reading being blinded. To achieve a high consistency, the examiner repeated the measurements until a high degree of agreement between the readings of the same set is achieved. Further, two-way mixed model intraclass correlation co-efficient (ICC) test was performed to check intra-examiner reliabilities. ICCs ranged from 0.900 to 0.997, which were interpreted as highly calibrated. [22]

### Statistical analysis

All statistical analyses were performed using SPSS software, version 25 (Chicago, IL, USA) and SAS version 9.4 (SAS Institute Inc., Cary, NC). Descriptive statistics (frequencies, means, standard deviation and standard error) were conducted. Although the crestal soft tissue thickness and the hard tissue measurements were slightly right skewed, our sample size was large enough for us to employ linear mixed models. Since some participants had multiple implants, linear mixed models were used to compare the crestal soft tissue thickness and the hard tissue measurements by gender, age groups, ethnic groups, and edentulous sites. Pearson correlation was applied to evaluate correlation between crestal soft tissue thickness and different hard tissue measurements while taking into account of repeated measures [23]. The association between crestal soft tissue thickness and the independent variables (gender, age groups, site groups) was evaluated using repeated measure logistic regression, while the crestal soft tissue thickness was dichotomized by a threshold of 2.0 mm. Odds ratios and 95% confidence intervals were estimated. Ethnicity was excluded from the logistic model due to its lack of variation. The statistical significance level was set to *p* < 0.05.

## Results

A total of 267 patients (138 female and 129 male) with 321 edentulous sites were included. Among those sites, 231 sites did not have any bone grafting procedure done (non-grafted native bone sites), while the remaining 91 sites had bone grafting procedure done so only soft tissue parameter could be measured (grafted sites). The mean age of the patients was 60 years (standard deviation 13.8, range 21–85 years). The majority of the patients (85.4%) were not Hispanic or Latino, eight patients were Hispanic or Latino, thirty patients did not report their ethnicity. 161 of the edentulous sites were maxillary posterior sites, 110 sites were mandibular posterior sites, 50 sites were anterior sites (incisors and canines) including 48 maxillary anterior sites and only 2 mandibular anterior sties (Table 1). The mean time patients have been in a status of edentulism was 282 days. More than 76% sites have been edentulous for longer than 90 days before CBCT was taken. Only 17 patients had removable temporary prostheses (Essix retainer or interim resin partial denture), and the mean duration of wearing the temporary prostheses before CBCT of the site taken was 152 days.

**Table 1:** Characteristics of patients (n = 267) and 321 edentulous sites

Variables	All sites [total number (%)]	Non-grafted native bone sites [total number (%)]	Bone-grafted sites [Total number (%)]
Gender			
Female	138 (51.7)	115 (49.8)	49 (54.4)
Male	129 (48.3)	116 (50.2)	41 (45.6)
Age [mean (SD)]	60.0 (13.8)	60.1 (13.4)	59.9 (15.2)
Age group			
18–40	25 (9.4)	20 (8.7)	12 (13.3)
41–65	132 (49.4)	115 (49.8)	41 (45.6)
> 65	110 (41.2)	96 (41.6)	37 (41.1)
Ethnic group			
Not Hispanic or Latino	229 (85.8)	196 (84.9)	75 (83.3)
Hispanic or Latino	8 (3.0)	8 (3.5)	1 (1.1)
No response	30 (11.2)	27 (11.7)	14 (15.6)
Edentulous site group			
Maxillary and Mandibular anterior sites	50 (15.6)	21 (9.1)	29 (32.2)
Maxillary posterior sites	161 (50.2)	123 (53.3)	38 (42.2)
Mandibular posterior sites	110 (34.3)	87 (37.7)	23 (25.6)
Days in a status of edentulism [mean (SD)]	282 (331)	318 (363)	190 (206)
Days wearing temporary prostheses [mean (SD)]	152 (120)	109 (110)	190 (122)

*SD* Standard deviation

In the 321 edentulous sites, the mean crestal soft tissue thickness was 2.16 ± 0.74 mm and the median thickness was 2.05 mm, ranging from 0.62 to 4.79 mm. Linear mixed models revealed the crestal soft tissue thickness was significantly different among edentulous site groups (*p* < 0.001) (Table 2). Among all sites, the crestal soft tissue thickness of anterior sites (mean = 2.41, SE = 0.105, *p* < 0.001) was significantly more than that of maxillary posterior sites (mean = 2.16, SE = 0.059) and mandibular posterior sites (mean = 2.06, SE = 0.07). Comparisons of crestal soft tissue thickness in non-grafted native bone sites and bone-grafted sites were also conducted. In non-grafted sites, anterior sites showed significantly thicker crestal soft tissue than posterior sites while in bone-grafted sites no significant difference was detected. Although among non-grafted sites, male subjects had statistically thicker crestal soft tissue than female subjects, the actual difference between the measurements was relatively small considering their clinical relevance. Crestal soft tissue thickness among different age groups did not show significant difference.

**Table 2:** Estimated means and SEs of thickness of crestal soft tissue at the alveolar crest

Demographics	Group	All sites (mm)	Non-grafted native bone sites (mm)	Bone-grafted sites (mm)
All		2.16 (0.044)	2.17 (0.048)	2.15 (0.091)
Gender	Female	2.09 (0.061)	2.04 (0.067)*	2.21 (0.124)
	Male	2.24 (0.063)	2.29 (0.068)*	2.08 (0.136)
Age group	18–40	2.37 (0.143)	2.46 (0.166)	2.2 (0.274)
	41–65	2.13 (0.063)	2.11 (0.067)	2.19 (0.134)
	> 65	2.16 (0.069)	2.17 (0.074)	2.1 (0.143)
Edentulous site group	Maxillary and mandibular anterior sites	2.41 (0.105)*	2.49 (0.152)*	2.41 (0.158)
	Maxillary posterior sites	2.16 (0.059)*	2.18 (0.063)*	2.03 (0.136)
	Mandibular posterior sites	2.06 (0.07)*	2.06 (0.075)*	2.04 (0.173)

*SE* Standard error

*: *p* < 0.05

For the non-grafted native bone sites, the repeated measure logistic regression model testing the association between thin or thick crestal soft tissue thickness (2.0 mm as the threshold) and the independent variables (gender, age groups, edentulous site groups) showed anterior sites [OR = 3.429 (1.100–10.69)] and maxillary posterior sites [OR = 1.937 (1.077–3.482)] had higher odds of presenting with ≥ 2.0 mm thickness of soft tissue at the alveolar crest compared to mandibular posterior sites (Table 3).

**Table 3:** Association between crestal soft tissue thickness and gender, age, and edentulous sites in non-grafted native bone sites using logistic regression

	Crestal soft tissue thickness N (%)		Crude odds ratio	
	< 2.0 mm	≥ 2.0 mm	Odds ratio (CI 95%)	*p-*value
Gender				0.258
Female	52 (45.2)	63 (54.8)	Ref	
Male	44 (37.9)	72 (62.1)	1.387 (0.786, 2.449)	0.258
Age group				0.729
18–40	8 (40.0)	12 (60.0)	Ref	
41–65	51 (44.4)	64 (55.7)	0.833 (0.291, 2.385)	0.732
> 65	37 (38.5)	59 (61.5)	1.055 (0.361, 3.077)	0.922
Edentulous site group				0.027
Mandibular posterior sites	46 (52.9)	41 (47.1)	Ref	
Maxillary posterior sites	45 (36.6)	78 (63.4)	1.937 (1.077, 3.482)	0.027*
Incisors and canines	5 (23.8)	16 (76.2)	3.429 (1.100, 10.69)	0.034*

*CI* Confidence interval

*Modeling the probability of having thick (≥ 2 mm) crestal soft tissue, *p* < 0.05

Among the 231 sites without any hard tissue grafting procedure, the mean thickness of cortical bone at the alveolar crest was 0.94 mm (SE = 0.041). Thickness of buccal and lingual cortical plates 5 mm apical to the alveolar crest were 1.17 mm (SE = 0.048) and 1.58 mm (SE = 0.055) respectively. Mean width of alveolar ridge (from buccal cortical plate to lingual cortical plate) 5 mm apical to the alveolar crest was 9.42 mm (SE = 0.173). Linear mixed models revealed the thickness of cortical bone at the alveolar crest, thickness of buccal cortical plate, lingual cortical plate and the width of alveolar ridge were significantly different among edentulous site groups (Table 4). Mandibular posterior sites had significantly more thickness of cortical bone at the alveolar crest (mean = 1.08, SE = 0.065, *p* < 0.05), thickness of buccal cortical plate (mean = 1.62, SE = 0.066, *p* < 0.001), thickness of lingual plate (mean = 2.19, SE = 0.071, *p* < 0.001), and width of alveolar ridge (mean = 10.46, SE = 0.244, *p* < 0.001) than maxillary posterior sites and anterior sites (maxillary and mandibular incisors and canines). However, if combing crestal soft tissue and crestal cortical bone as a whole unit, the thickness of this biological unit did not show significant difference among edentulous site groups. The result also showed male patients’ hard tissue measurements were significantly larger than female patients’, except the thickness of cortical bone at the alveolar crest. Hard tissue measurements among different age groups did not show statistical significance. Combing thickness of crestal soft tissue and crestal cortical bone as a new parameter, male patients measured significantly thicker. Since a sizable proportion (> 85%) of the patients were from the same ethnic group, comparison of crestal soft tissue thickness and hard tissue measurements among different ethnic groups, and association between ethnicity and clinical parameters were not analyzed.

**Table 4:** Estimated means and SEs of hard tissue measurements in non-grafted native bone sites

Demographics	Group	Thickness of cortical bone at the alveolar crest (mm)	Thickness of buccal cortical plate 5 mm apical to the alveolar crest (mm)	Thickness of lingual cortical plate 5 mm apical to the alveolar crest (mm)	Width of alveolar ridge 5 mm apical to the alveolar crest (mm)	Thickness of crestal soft tissue and crestal cortical bone
All		0.94 (0.041)	1.17 (0.048)	1.58 (0.055)	9.42 (0.173)	3.10 (0.063)
Gender	Female	0.89 (0.058)	1.03 (0.065)**	1.44 (0.077)**	8.89 (0.236)**	2.94 (0.088)**
	Male	0.99 (0.059)	1.33 (0.067)**	1.73 (0.079)**	9.99 (0.242)**	3.27 (0.09)**
Age group	18–40	0.83 (0.144)	1.19 (0.164)	1.49 (0.193)	9.29 (0.604)	3.3 (0.218)
	41–65	0.95 (0.059)	1.09 (0.068)	1.61 (0.08)	9.5 (0.249)	3.05 (0.091)
	> 65	0.95 (0.065)	1.27 (0.073)	1.56 (0.086)	9.38 (0.269)	3.12 (0.098)
Edentulous site group	Maxillary and Mandibular Anterior sites	0.88 (0.132)*	0.85 (0.13)***	1.00 (0.14)***	6.93 (0.486)***	3.47 (0.202)
	Maxillary Posterior sites	0.85 (0.055)*	0.91 (0.056)***	1.24 (0.06)***	9.05 (0.209)***	3.02 (0.086)
	Mandibular Posterior sites	1.08 (0.065)*	1.62 (0.066)***	2.19 (0.071)***	10.46 (0.244)***	3.13 (0.101)

*SE* Standard error

*: *p* < 0.05; **: *p* < 0.01; ***: *p* < 0.001

Pearson’s correlation showed moderate positive correlation among hard tissue measurements, but weak correlation between crestal soft tissue thickness and hard tissue measurements (Table 5). Thickness of buccal cortical plate 5 mm apical to the alveolar crest was positively correlated with thickness of cortical bone at the alveolar crest, thickness of lingual cortical plate 5 mm apical to the alveolar crest, and width of alveolar ridge 5 mm apical to the alveolar crest. Thickness of lingual cortical plate 5 mm apical to the alveolar crest was positively correlated with thickness of cortical bone at the alveolar crest, and width of alveolar ridge 5 mm apical to the alveolar crest.

**Table 5:** Pearson’s correlation between vertical soft tissue thickness and different hard tissues measurements in non-grafted native bone sites

	Thickness of soft tissue at the alveolar crest	Thickness of cortical bone at the alveolar crest	Thickness of buccal cortical plate 5 mm apical to the alveolar crest	Thickness of lingual cortical plate 5 mm apical to the alveolar crest
Thickness of cortical bone at the alveolar crest	0.02			
Thickness of buccal cortical plate 5 mm apical to the alveolar crest	− 0.05	0.40*		
Thickness of lingual cortical plate 5 mm apical to the alveolar crest	− 0.06	0.29*	0.56*	
Width of alveolar ridge 5 mm apical to the alveolar crest	0.02	− 0.01	0.44*	0.41*

*Correlation is significant at the 0.001 level (2-tailed)

## Discussion

The crestal soft tissue thickness of 321 sites planned for implant placement was evaluated in the present study. The mean time of the sites having been in a status of edentulism was 282 days and majority of the sites had been healed for longer than 3 months before CBCT was taken. Very few patients (6%) had temporary prostheses during the edentulous phase and only removable prostheses were used. Taken these factors into consideration, we could say most sites were matured and healed without pressure from temporary prosthese when being evaluated in this study. If we use 2.0 mm as a cutoff point to divide between thin and thick tissue as in some other clinical studies [13, 24], the current study indicated that about 50% of the population had relatively thick crestal soft tissue no matter whether or not hard tissue grafting procedure was conducted. Linkevic̆ius [25] estimated that 30–80% of patients might have thin crestal soft tissue, but the range was wide, and the estimation was not supported by any epidemiologic study. One previous systematic review compared mean crestal soft tissue thicknesses reported in different clinical studies. The thinnest mean tissue thickness reported was 1.53 ± 0.07 mm, and the thickest mean tissue thickness reported was 3.32 ± 0.76 mm. [12] The mean crestal soft tissue thickness measured in the current study (2.16 ± 0.74 mm) fell between those two groups. Our study also showed there was no significant difference of crestal soft tissue thickness between sites received bone grafting procedure and sites did not. Systematic reviews of clinical studies have concluded that there is moderate certainty of the evidence that implants placed with an initially thicker peri-implant soft tissue (or supracrestal tissue attachment) have less radiographic marginal bone loss in the short term [12, 26]. In contrast, one recent clinical study found excessive vertical soft tissue thickness around implants in patients with a history of periodontitis had an adverse influence on health of the peri-implant tissue [27]. The mean crestal soft tissues reported in that study was 3.74 ± 1.50 mm. The current study was the first one that reported the variations of crestal soft tissue thickness using non-invasive radiographic method, indicating more than half of implant patients may have relatively thick initial crestal soft tissue, and bone grafting procedure might not affect the crestal soft tissue thickness. The clinical significance of crestal soft tissue to long-term health of peri-implant tissue is still inconclusive.

The results of our study showed that the thickness of crestal soft tissue of non-grafted native bone sites varied among different edentulous site groups, with anterior sites (predominantly maxillary incisors and canines) demonstrating greater values than those of posterior sites. The mean crestal soft tissue thickness of anterior sites was 2.49 mm. While mean crestal soft tissue thicknesses of maxillary posterior sties and mandibular posterior sites were 2.18 mm and 2.06 mm, respectively. Anterior sites and maxillary posterior sites also had higher chances of presenting ≥ 2.0 mm thickness of soft tissue at alveolar crest than mandibular posterior sties. To our knowledge, no previous study has reported the differences of thickness of crestal soft tissue between different sites. Since the present study did not measure the attachment level or soft tissue thickness of adjacent teeth, it was hard to explain the exact reason why mandibular posterior sites showed significantly thinner crestal soft tissue. The non-grafted anterior sites (21 sites) included in this study were relatively small compared with the number of non-grafted maxillary and mandibular posterior sites. More studies and larger sample sizes are needed to confirm this finding.

Hard tissue parameters were analyzed in the present study using data collected from 231 sites that did not undergo any bone grafting procedure. Previous studies evaluated buccal bone thickness and have found a thin (< 1.0 mm) bone wall usually presented in anterior regions of both jaws [28–30]. Several studies have focused on the thickness of the buccal cortical plate and its correlation between facial tissue thickness, especially in the anterior area. Younes et al. [31] studied the relationship between buccal bone and soft tissue thickness at teeth in the premaxilla. They included 21 patients, measured buccal bone thickness on CBCT scans and used an ultrasonic device to measure the gingival thickness. Their results showed a thin buccal bone wall (< 1.0 mm) in over half of the central incisors and canines, and there was a moderately positive correlation between buccal bone and soft tissue thickness. Esfahanizadeh et al. measured buccal bone and soft tissue thicknesses of 330 maxillary incisors using CBCT scans and their results were consistent with Younes et al. They reported that the mean thickness of buccal bone and soft tissue in the anterior maxilla was < 1.0 mm and there was a mild linear correlation between them [32]. Fu et al. found that the mean labial soft tissue thickness of the maxillary anterior teeth at 2.0 mm below the bone crest was 0.57 mm (0.2–1.86 mm) on CBCT scans and the thickness of the gingival tissue had a moderate association with the underlying labial alveolar bone [33]. In contrast, Kim et al. found that the correlation between buccal bone thickness and soft tissue thickness of anterior teeth was generally not significant [34]. Most of the studies mentioned above measured the facial bone wall thickness at different levels from the bone crest. Among the different levels, 5 mm apical to the bone crest was selected in all of these studies. Another study reported that the mean values of facial bone thickness for the different levels fluctuated in a very small range [35]. Because the primary focus of our study was crestal soft tissue thickness, we decided only to evaluate the thickness of buccal plates 5 mm apical to the alveolar crest. In the 231 sites from our study, the mean thickness of buccal cortical plate was 1.17 mm, indicating relative thick (> 1.0 mm) buccal cortical plates existed in sites that were ready for implant placement. Male patients and mandibular posterior sites had significantly thicker buccal cortical plates. However, most sites from the present study were posterior sites, and previous studies reported thickness of bone around teeth while our study only focused on edentulous sites. It is difficult to compare the results of the present study with other studies.

Crestal cortical bone thickness and lingual cortical plate thickness 5 mm apical to the alveolar crest were also measured in this study. Our study found a significant difference in crestal cortical bone thicknesses among different site groups with mandibular posterior sites measuring the thickest and maxillary posterior sites measuring the thinnest. Ko et al. measured 661 implant sites using CBCT and reported that the crestal cortical bone thicknesses at dental implant sites in the four regions decreased in the following order: posterior mandible (1.07 ± 0.47 mm) > anterior mandible (0.99 ± 0.36) > anterior maxilla (0.82 ± 0.30 mm) > posterior maxilla (0.75 ± 0.35 mm) [17]. Another study measured 218 patients’ CBCT scans of implant sites, and reported the thickness of crestal cortical bone in the posterior mandible, anterior mandible, anterior maxilla, and posterior maxilla being 1.18 ± 0.48, 1.08 ± 0.30, 0.82 ± 0.32, and 0.76 ± 0.28 mm, respectively. [36] Although our study grouped maxillary and mandibular anterior sites together due to limited sample size, the findings were considered consistent with the previous studies. The present study found that the thickness of the lingual cortical plate and width of alveolar ridge decreased in the following order: posterior mandible > posterior maxilla > anterior. The mean thickness of the lingual cortical plate was 1.58 mm, which was consistent with the finding reported by Srebrzyńska-Witek et al. [37] They measured 100 CBCT scans of anterior mandible and found the mean thickness of the lingual alveolar cortex was 1.51 mm ± 0.35 mm.

According to our results, most of the hard tissue measurements were positively correlated to each other while the correlation between hard tissue measurements and crestal soft tissue thickness was not significant. One review reported no significant correlation had been found between facial soft tissue thickness and underlying buccal bone thickness according to several previous studies [38]. Findings from the present study indicated that there might not be significant correlation between crestal soft thickness and underlying crestal bone thickness either. However, when we consider crestal soft tissue and cortical bone at the alveolar crest as a biological unit, our study showed there might be a trend that the total thickness could be relatively constant. More investigation will be needed to evaluate the correlation between hard tissue measurements, crestal soft tissue thickness, and buccal soft tissue thickness.

CBCT scans were used in this study to measure crestal soft tissue thickness. Even though the soft tissue of the lips and cheeks were not retracted when taking CBCTs, and the lips and cheeks did collapse on the buccal gingiva in most cases, a clear visualization of the crestal soft tissue was achieved in most cases. The advantages of CBCT included high resolution, more precision in linear measurements of both soft and hard tissues than traditional X-rays, and measurement of tissue thickness prior to the surgical procedure. Fu et al. did not find statistically significant differences between the clinical and CBCT measurements of both soft tissue and bone thickness when measuring tissue biotypes [33]. The current study also indicated that CBCT can be used as an objective and non-invasive method to assess crestal soft tissue thickness.

The present study has some limitations. One limitation is the small sample size of anterior sites. We had to combine maxillary and mandibular anterior sites together into one group in order to compare the measurements to posterior groups. Additional studies are needed to compare the crestal soft tissue thickness between maxillary and mandibular anterior sites. The present study only focused on the edentulous sites and did not measure the periodontal condition of adjacent teeth. Studies that included both edentulous sites and adjacent teeth may be needed to evaluate the effects of periodontal condition of adjacent teeth to the edentulous sites. The present retrospective study evaluated the correlation between crestal soft tissue thickness and hard tissue measurements but did not evaluate buccal soft tissue thickness and its relationship to crestal soft tissue. Further prospective studies with a larger sample size and longer follow-up period are needed to investigate crestal soft tissue changes before and after implant placement and its long-term effect on implant success.

## Conclusion

According to the results of our study, crestal soft tissue of more than half of the edentulous sites which were ready to receive implant placement was found to be relatively thick (> 2.0 mm). Crestal soft tissue thickness was not significantly associated with hard tissue measurements. Edentulous anterior sites and maxillary posterior sites may present with thicker soft tissue at the alveolar crest than mandibular posterior sties. Gender and age did not appear to have a significant influence on crestal soft tissue thickness.

## Data Availability

The datasets used during the current study are available from the corresponding author on reasonable request. All data analyzed during this study are included in this published article in the form of tables and figures.

## Abbreviations

CBCT: Cone beam computed tomography
FOV: Field of view
ICC: Intraclass correlation co-efficient
SE: Standard error
OR: Odds ratio
CI: Confidence interval
